# Novel SARS-CoV-2 therapeutic targets: RNA proofreading complex and virus-induced senescence

**DOI:** 10.1038/s41418-021-00909-6

**Published:** 2022-01-17

**Authors:** Liangyu Lin, Ying Wang, Qing Li, Mingyuan Hu, Yufang Shi

**Affiliations:** 1 CAS Key Laboratory of Tissue Microenvironment and Tumor, Shanghai Institute of Nutrition and Health, University of Chinese Academy of Sciences, Chinese Academy of Sciences, 320 Yueyang Road, Shanghai, 200030 China; 2 The First Affiliated Hospital of Soochow University, State Key Laboratory of Radiation Medicine and Protection, Institutes for Translational Medicine, Soochow University Medical College, Suzhou, Jiangsu 215123 China; 3 Department of Experimental Medicine, TOR, University of Rome Tor Vergata, Rome, 00133 Italy

**Keywords:** Infectious diseases, Respiratory tract diseases

Severe acute respiratory syndrome coronavirus 2 (SARS-CoV-2) is the causative viral pathogen of the coronavirus disease 2019 (COVID-19), which has led to 250 million infections and more than 5.00 million deaths worldwide by the middle of October, 2021 (WHO). Although available vaccines can lower the risk of developing symptomatic COVID-19, the world is still under the threat of SARS-CoV-2 due to the lack of highly effective treatments [1, 2]. Suppressing intracellular viral replication and eliminating the infected cells are the two major strategies to limit the severities of SARS-CoV-2 infections. However, little success has been achieved and novel efficient therapeutic targets are yet to be identified. Two latest papers published in *Cell Death and Differentiation* [3] and *Nature* [4] showed that coronavirus RNA repair complex NSP14/NSP10 and SARS-CoV-2-induced cellular senescence are druggable targets for SARS-CoV-2 infections. Thus, SARS-CoV-2 RNA repair complex inhibitor sofalcone and senolytics could be applied to treat COVID-19 infections.

SARS-CoV-2 is single-stranded positive-sense RNA virus belong to the β-coronavirus genus [5], able to attack the immune system [6, 7]. Compared to other RNA virus, coronavirus have dramatically larger genomes (~30 kb). To maintain the integrity of their genome and prevent lethal mutagenesis, coronavirus developed a specialized RNA proofreading mechanism that, excises misincorporated nucleotides from the nascent RNA [8]. Such effects were mediated by the NSP14/NSP10 complex containing an exonuclease domain. The RNA proofreading complex not only ensured the replication fidelity but also impaired the therapeutic effects of anti-virus agents, especially nucleotide analogs, which are incorporated into the viral genome to induce premature replication termination [9].

Currently, remdesivir is the only anti-viral drug approved by FDA for the treatment of hospitalized COVID-19 patients. According to the phase III clinical trial report, remdesivir moderately speeds up the recovery of COVID-19 patients [10]. Such therapeutic efficacy does not fully meet the urgent medical demands imposed by SARS-CoV-2 infections and the approval of remdesivir also raised debates among the scientific community. It is important to note that most nucleotide analogs stop viral replication once they are incorporated into the viral genome. The incorporation of remdesivir, an adenosine nucleotide analog, could allow up to three correct nucleotides insertion into the virus genome [11]. Loss-of-function mutation of NSP14 in coronavirus significantly sensitized the virus to remdesivir, suggesting the existence of a drug resistance mechanism mediated by NSP14 [12]. The example set by remdesivir suggests that targeting the RNA proofreading complex NSP14/NSP10 may unlash the therapeutic effects of nucleotide analogs, which are the largest class of anti-virus drugs [13].

Considering the beneficial effects of targeting RNA proofreading complex in control coronavirus, it is imperative to develop specific inhibitors targeting NSP14/NSP10. However, this is challenging since NSP14/NSP10 exonuclease activities are measured using gel-based assay, which is not compatible with large-scale screening of compound libraries. To overcome this obstacle, Rona et al. established a new assay modified from fluorescence resonance energy transfer (FRET) to determine the activities of NSP14/NSP10. Briefly, dsRNA with very low *T*_m_ were labeled with a fluorophore and a quencher. The exonuclease activities of NSP14/NSP10 recognize and remove the mismatched base pairs and eventually separate the two RNA strands, thus dissociating the quencher from the fluorophore. Therefore, the catalytic activities of NSP14/NSP10 exonuclease could be obtained by monitoring the changes of fluorescent intensity. After pre-selection by an in silico screen, 122 compounds were tested with the FRET system to evaluate their inhibitory effects on NSP14/NSP10. The identified NSP14/NSP10 inhibitors with low micromolar IC_50_ were further evaluated using HCoV-OC43 and SARS-CoV-2 infected cells individually or in combination with remdesivir. None of identified NSP14/NSP10 inhibitors alone could suppress the replication of HCoV-OC43 and SARS-CoV-2. However, three NSP14/NSP10 inhibitors showed synergistic effects with remdesivir to inhibit coronavirus replication in infected cells. The most potent identified inhibitor is sofalcone, which lowered the IC_50_ of remdesivir by ~5 fold. Sofalcone is an oral gastrointestinal medication used in Japan with validated safety [14], and this is expected to accelerate the translation of the current findings.

The replication of SARS-CoV-2 is the “root of all evil.” However, what makes COVID-19 a deadly disease is the overwhelming immune responses triggered by the virus infected cells. Therefore, strategies that accelerate the clearance of virus-infected cells could also be considered for COVID-19 treatment, especially for patients suffering from severe COVID-19. *Soyoung* Lee et al. showed that the SARS-CoV-2 infection could induce typical cellular senescence phenotypes both in in vitro infected human diploid fibroblast and in SARS-CoV-2 lung sections [4]. Such virus-induced senescence (VIS) is indistinguishable from other forms of cellular senescence and is accompanied by a senescence-associated secretory phenotype (SASP) which drives the activation of macrophages. Taking advantage of the similarities between VIS and the well-studied types of senescence, the authors employed the currently available senolytics to eliminate the virus-infected cells. It was found that these senescence cells are indeed sensitive to senolytics such as Bcl-2 inhibitor Navitoclax and multiple kinases-inhibiting flavonoids Fisetin and Quercetin. Furthermore, oral administration of these senolytics to animal model of COVID-19 infections reduced systemic inflammation and mitigated the diseases.

The evolution of SARS-CoV-2 variants is now becoming a new challenge for controlling the COVID-19 pandemic. Combined strategies including blocking the entry of virus, suppressing virus intracellular replication, and deleting the virus-infected cells should be considered during the development of treatments against COVID-19 (Fig. 1).

**Fig. 1:**
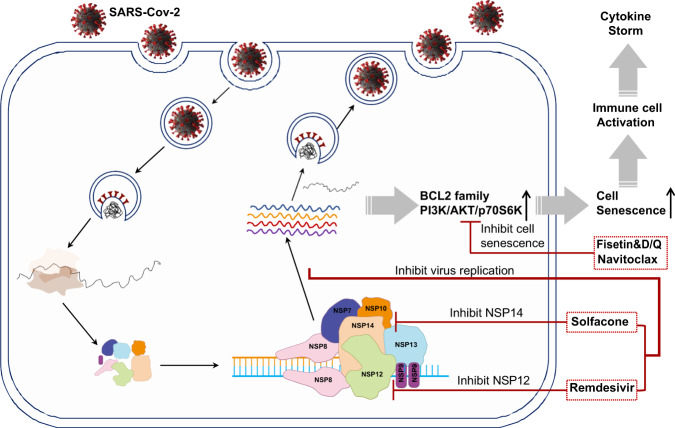
Viral and cellular targets of SARS-CoV-2. The intracellular replication of SARS-CoV-2 is mediated by the viral RNA-dependent RNA polymerase (NSP12). The incorporation of remdesivir terminates the assemble of viral genomes. However, the efficacy of remdesivir could be compromised by RNA proofreading complex (NSP10/NSP14), Blocking NSP14 activity by solfacone dramatically lowered the IC50 of remdesivir. The continuous intracellular virus replication would induce typical cellular senescence with the induction of anti-apoptotic Bcl-2 family and PI3K signaling cascade. Senolytics, such as fisetin and quercetin, could help to clear infected cells and resolve inflammation in COVID-19 infections.

## Supplementary information

The Author Contribution Form
