# Supplementary material for: Novel SARS-CoV-2 therapeutic targets: RNA proofreading complex and virus-induced senescence
Source: Cell Death Differ. 2022 Jan 17;29(2):263–5. doi: 10.1038/s41418-021-00909-6 (PMC8761837; doi:10.1038/s41418-021-00909-6)
Supplement: Supplementary file 1 — The Author Contribution Form [file 41418_2021_909_MOESM1_ESM.pdf]

DECLARATION OF CONTRIBUTIONS TO ARTICLE

ADMC

Manuscript Number: CDD-21-2527

Journal Name: *Cell Death & Differentiation* (the 'Journal')

Proposed Title of the Contribution:

Novel SARS-CoV-2 Therapeutic Targets: RNA Proofreading Complex and Virus-induced Senescence (the 'Contribution)

Author(s):

Liangyu Lin, Ying Wang, Qing Li, Mingyuan Hu, and Yufang Shi (the 'Authors')

For all *CDD* articles, each person named as an author in the published version must be able to show he or she has contributed substantially to the article.

Authorship credit should be based on 1) substantial contributions to conception and design, acquisition of data, or analysis and interpretation of data; 2) drafting the article or revising it critically for important intellectual content; and 3) final approval of the version to be published. Authors should meet conditions 1, 2 and 3.

Any person who cannot be shown to have made a substantial contribution to the article cannot be listed as an author in the final version. The name of any person who is deemed to have made a minor contribution can, however, appear in the Acknowledgments section of the article.

Please complete the table below to indicate the contributions of all named authors to the manuscript.

Author Full Name:	Specification of Contribution to the Manuscript:
Liangyu Lin	Conceptional design and manuscript drafting
Qing Li	Conceptional design and figure construction
Mingyuan Hu	Conceptional design and manuscript drafting
Ying Wang	Conceptional design and manuscript revision
Yufang Shi	Manuscript drafting and the final approval of the paper

Please complete the table below to indicate the contributions of all named authors to the figures.

Figure 1:

Liangyu Lin, Qing Li, and Yufang Shi

Figure 2:

Figure 3:

Figure 4:

Figure 5:

Figure 6:

Signed for and on behalf of the Author(s):	Print Name:	Date:
	Yufang Shi	2021.11.17
